# Ten simple rules for providing a meaningful research experience to high school students

**DOI:** 10.1371/journal.pcbi.1006920

**Published:** 2019-04-25

**Authors:** Emily A. Lescak, Kate M. O’Neill, Giovanna M. Collu, Subhamoy Das

**Affiliations:** 1 Department of Biological Sciences, University of Alaska Anchorage, Anchorage, Alaska, United States of America; 2 Institute for Physical Science and Technology, University of Maryland, College Park, Maryland, United States of America; 3 National Institute of Neurological Disorders and Stroke, National Institutes of Health, Bethesda, Maryland, United States of America; 4 Department of Cell, Developmental and Regenerative Biology and Graduate School of Biomedical Sciences, Icahn School of Medicine at Mount Sinai, New York, New York, United States of America; 5 Department of Neurosurgery, Stanford School of Medicine, Stanford, California, United States of America; Whitehead Institute for Biomedical Research, UNITED STATES

## Introduction

Much has been written about designing research experiences for undergraduate students [1–4], but what about providing meaningful experiences to high school students? There are many formal opportunities for high school students to conduct research, but early-career scientists and principal investigators (PIs) do not necessarily have much experience working with this age group, which presents different opportunities and challenges than working with undergraduates. Thus, we present guidance in this Ten Simple Rules article on how to be an effective research mentor for high school students based on our experiences as early-career biologists and our formal mentor training.

Studies show that students—and the general public as a whole—have a narrow view of what a scientist is, does, and looks like [5, 6]. The opportunity to work in a research group may be the first time that high school students encounter a “real scientist.” Likely, it is also their first chance to peek inside the black box that is scientific research—something they may only know from the media. They will experience firsthand what it is like to work in a research environment (whether they are doing experiments or computational work) and will likely be surprised by how communication and collaboration not only are necessary to the scientific process but also make research more rewarding. Performing scientific research gives students the opportunity to witness the practical applications of concepts they have been taught in school and to observe how the experimental and analytical work done in research settings builds upon what they have learned in the classroom. Importantly, they will also experience the excitement and challenges of investigating open-ended questions without predetermined answers. Authentic research experiences can empower students to pursue research opportunities as undergraduates and to consider careers in science, technology, engineering, and mathematics (STEM).

Engaging high school students in research and the process of doing science allows them to form meaningful relationships with mentors who can help them stay on track academically, serve as role models, and help prepare them for future careers. By working with high school students from the local community, mentors can bridge the gap between scientists and the general public and encourage students to attend their local university, which is a benefit for the mentor’s institution, too. For high school students—particularly those who will be first-generation college students—getting comfortable on their local college campuses can make a meaningful impact on their educational goals. There are also opportunities for their supervisors, who are often early-career scientists (graduate students or postdoctoral fellows [postdocs]), to broaden their mentoring skills, improve their communication of the complexities of everyday science to a new audience, and learn how to develop tangible project goals that can be tackled within a finite period—all of which are excellent professional development opportunities.

Opportunities for high school students may be initiated either informally, through outreach with local schools, or formally, through an established program. We have compiled a list of programs, organized by state, that provide high school students with research experiences; please note that this list is not exhaustive. In general, placements range from the occasional half-day visit to year-long internships, and some placements are not necessarily local. Although the rules presented here are intended to guide mentors who will work with students for at least a few weeks, mentors working with students for shorter periods may also find some of these rules helpful.

Some universities and medical schools have volunteer offices or organized programs for bringing high school students into the laboratory, so check whether there are already connections to schools in your area through previous student placements. Moreover, when initiating contact with prospective mentees, consider the opportunity you have to make a meaningful impact in the lives of young people who come from historically underrepresented and underserved populations or underprivileged backgrounds. Scientific societies and funding agencies may have specific mechanisms for funding summer high school students, and many of these are intended for students who come from groups that are underrepresented in science. Example programs from the list above include the American Fisheries Society’s Hutton Program and the Short-Term Experience for Underrepresented Persons at the National Institute of Diabetes and Digestive and Kidney Diseases. Some of these programs also provide stipends for the students, which relieves the additional pressure of needing to find a summer job. However you decide to bring a high school student into the laboratory, be sure to discuss with the prospective mentee what they hope to gain from the experience to make sure that your expectations are aligned before either of you commits to the placement.

It is important to recognize that working with high school students presents different challenges and opportunities than working with undergraduates. For example, high school students may be more enthusiastic than undergraduates about performing research because they have likely only engaged in simple lab exercises at school. However, they also have less scientific knowledge than undergraduates and likely are not able to spend as much time doing research because of schedule restrictions. These challenges can easily be mitigated by the mentor with some planning, and we have found mentoring high school students to be extremely rewarding.

If you decide to take on a high school student, we offer ten simple rules as guidance for providing the student with a positive experience while they are working with you. Although these rules were written with postdocs and advanced graduate students as the intended audience, we anticipate that they will also be helpful for PIs who have not yet hosted a high school student in their lab. In addition to these rules, we also recommend participating in mentor training through the National Research Mentoring Network or a similar program and familiarizing yourself with the literature on best practices in mentoring ([7–9] among many others) to strengthen your foundation in communicating and goal setting.

## Rule 1: Check with your institution’s environmental health and safety/risk management offices to confirm the rules and regulations for working with minors

Anyone working in a research lab must be compliant with institutional safety regulations. It is important to be fully aware of the required paperwork, training, permissions, and other administrative steps before you reach out to potential mentees. Students performing computational work will likely still have some training to complete before they can begin working in the lab. As the primary mentor, it is your job to work with your PI and institutional officials (e.g., environmental health and safety officers, building managers) to find out what needs to be done for you to be able to work with the student and for the student to be able to work in the lab.

Any online training that the student can complete before they start in the lab will save valuable research time, but they will likely have to participate in on-site training too. You as the mentor may also be required to complete specific training for working with minors, and the student’s guardian will likely need to sign consent forms. If feasible, schedule an initial, in-person meeting that includes the student, their guardian, the placement coordinator (if applicable), and the lab PI to explain the nature of the work and address any concerns. When the student does start working in the lab, and if they are doing experimental work, there might be protocols or procedures in which they cannot participate directly because of their status as a minor (e.g., working with vertebrates or using high-risk equipment), but you can involve them by allowing them to observe you during these tasks if it is safe and legal to do so. Make sure that you have personal protective equipment (lab coats, gloves) in the appropriate size. Finally, be sure to document any training you and the student complete and keep copies in your office and with the lab’s personnel records.

## Rule 2: Make sure that you and your PI agree on reasonable, time-bound expectations and goals for the student’s mentoring experience

Be proactive in planning the student’s placement in the lab, and discuss with your PI how you plan to manage your time with the student. Have a conversation with your PI and other lab members (if appropriate) about both the concerns associated with taking on a high school student and how the mentorship can benefit the lab’s research program, your professional development, and, of course, the prospective student! By taking these steps, you will ensure that the relationship between the student, PI, and other lab members is off to a good start and that you and your PI are on the same page regarding expectations for—and limitations of—the experience.

## Rule 3: Be realistic about your expectations for the student, and provide positive feedback

For the student to have a positive experience in the lab, it is important to set them up for success by designing a realistic project. You have to consider how much time they can actually commit to the project outside of school—and also how much time you have—and whether you will have a whole day with them or only smaller blocks of time. Student availability will vary depending on the kind of placement and its required time commitment. Discuss the student’s school curriculum with them or (if possible) their teachers to ensure that the project is designed at an appropriate level. A good project will result in the student feeling that they have accomplished something, learned new information and skills, and contributed to the lab’s larger goals by the end of their time with you.

An equally important aspect of your mentoring relationship is providing positive, constructive feedback. The student may not have confidence in their laboratory or analysis skills, because they will be new to research, so make sure to praise them for their work. Positive affirmations will help them gain confidence in their abilities, which is particularly important for women [10] and other groups underrepresented in STEM [11]. Inevitably, the student will make mistakes (we all do!), but make sure you highlight what they have done well. Then, together, brainstorm ways they can improve. These microaffirmations can go a long way in inspiring them to realize their own ability.

## Rule 4: Set goals early, and revisit them often

The student might have unrealistic expectations of what they can accomplish during their research experience because they are new to laboratory research. Thus, it is your duty as the mentor to explicitly set goals with both the best- and worst-case scenarios in mind and to manage expectations. We suggest you familiarize yourself with some of the resources available on goal setting [12, 13] to ensure that the goals you set for the student are realistic.

Set overall goals for the entire duration of the student’s research experience as well as for smaller periods of time (e.g., weekly), and revisit them regularly. The overall goal could be as simple as learning a new technique or analysis method or as complex as answering a small scientific question. High school students are generally accustomed to structured approaches in their schools, so providing a structured plan will help them to be productive in the lab and not feel overwhelmed. It will be a learning experience for them to realize that experiments or analyses often present technical difficulties and that original hypotheses are not always supported. You can show them how you iteratively improve, how you learn lessons from difficult experiments or analyses, and how these factors influence your goals. You can use these instances as teaching opportunities and explain that troubleshooting and course correction are critical steps in the scientific discovery process. Finally, to put everything on paper, consider developing a document in collaboration with the student that outlines expectations for communication and goals for your time together [14]. In this document, make sure that you agree with the student and guardian how you will communicate and that the student understands that they need to let you know in advance if they are unable to be in the lab at their scheduled time. This will help them develop their understanding of professional norms and managing deadlines.

## Rule 5: Design a deliverable for the end of the experience

Just like high school students have finals or class projects at the end of the term, it is important for you to work with the student to produce a final deliverable at the conclusion of the research experience. Examples of deliverables include the following: (1) a short summary to be shared with their teachers or school newspaper; (2) a presentation to their science class; (3) a summary for their college application; or for longer-term placements, (4) a poster presentation at the university/institution or a local conference. Formal mentorship programs likely require a presentation in one of these formats. Establish this expectation at the start of your time together (see Rule 4), and set aside time for the student to start working on the deliverable as soon as possible. Provide accessible, relevant background literature so that they can begin learning on their first day in the lab. To ensure they stay on track, set checkpoints along the way so that the student can complete the deliverable on time. If they are not part of a formal program that has a planned presentation at the end, discuss which of the various options works best for them. The experience of summarizing and presenting their research—no matter the format—is not only a valuable learning opportunity in terms of understanding their own work but also important for developing communication skills. Bear in mind that the student will need guidance in best practices for presenting and synthesizing their work. Provide them with examples and resources to empower them to be successful, and start the process early to avoid unnecessary stress.

## Rule 6: Structure the student’s time when they are in the lab

Do not assume that the student has experience with time management, because it is generally managed for them by their school. Spend time explaining how you plan your schedule and manage distractions. Encourage good practices, such as planning experiments or analyses in advance and filling in their lab notebook. For long-term placements (summer or year-long), be proactive about dividing your own time between working with the student and working on your independent research. Build in dedicated time for the student to read through protocols and any other information that you provide so that they can process and reinforce the knowledge they are acquiring. If the placement requires the student to spend several hours in a row working in the laboratory, pay attention to their energy levels, and be flexible about break times because they are likely accustomed to having breaks throughout the day at school.

To enhance the student's experience, also consider introducing them to other scientists and staff at your institution so that they can learn about different aspects of research and STEM careers. This can be transformative in college planning and can also expose them to career options they did not know existed. If you teach or engage in outreach or other aspects of service, consider allowing the student to attend your classes or meetings, if appropriate, so that they have a more complete idea of what a day in the life of a scientist is like.

## Rule 7: Help the student see both the forest and the trees

An important aspect of learning to think like a scientist is to understand the big picture (the forest) and how each experiment (the trees) meshes with those goals. Explain to the student the context of the project to which they are contributing, the big questions that you are trying to answer, and how their work fits into the lab’s overall goals. It can be easy for students to undervalue the work they do, particularly because day-to-day lab work tends to be iterative with incremental gains. Impress upon them the value of their work, and make sure they thoroughly understand each step. Consider also inviting the student to research group meetings so that they can better understand the broader picture of the work you are doing and the collaborative nature of research.

## Rule 8: Guide the student toward becoming independent in their work and taking ownership of their project

Performing scientific research in the laboratory requires a level of independence that is not as necessary in the classroom, and this may surprise the student working with you. To help them grow as a scientist, make sure to explain this difference at the beginning of your time together, and reinforce it often. Explain how, unlike projects designed for laboratory courses in school, there is no answer you “should” get in scientific research. There may be an answer that you are expecting—your hypothesis—but even the interpretation of those results can be open ended. Demonstrate to the student how you think outside the box when planning the next step or interpreting results, and encourage them to share their ideas. By brainstorming next steps together, you will teach the student by example how to take ownership of their project.

As their research progresses, hopefully the student is becoming proficient in experimental and/or analytical skills. Make sure that you are available when they are doing experiments and analyses, and be sure to guide them fully through a technique the first few times by showing them first and then doing it together until they feel comfortable. At this point, you will still need to supervise them to make sure they are working safely in the lab or setting up their analyses correctly. Build in reflective checkpoints so that the student can track their progress. They will likely have many questions at first and may not understand the purpose of each step—science is not always intuitive. As the student becomes more independent, ask them in an informal and nonintimidating way why they do a certain step in an experiment or analysis. When they do make a mistake, address it right away, and assure them that it is part of the learning process. Teach them how to document any errors and resulting mitigation through note-taking. You can impress upon the student the importance of taking detailed notes, but they will need guidance on how to keep a lab notebook [15]. You can help by checking their notebook regularly and providing feedback (see Rule 3). Explain how the documentation process is critical for reproducibility, and relate it back to the “lab reports” that they have done—or will do—in school.

## Rule 9: Show the student you are human

High school students may be intimidated by you or your lab-mates even though that is not the intent. To ensure that the student feels welcomed in the lab, make sure to introduce them to other lab members during lunch or during a regularly scheduled group meeting. To become more relatable to the student, have conversations with them about what you were like at their age, your hobbies and experiences at school, and how you got to where you are today. Sharing the challenges that you have overcome will help the student understand that they are not expected to be perfect. They are likely anxious about the possibility of making a mistake, ruining your experiment, or not making a good impression. Showing the student how you handle and learn from mistakes will take some of the pressure off them.

Also, allow the student to see that you have a life outside of your work. If you have a family or other caregiving responsibilities that you feel comfortable talking about, share them with the student. It can be transformative for students to see that scientists can manage personal and work responsibilities. Doing so will also humanize you and strengthen your ability to be a role model for a diverse range of students.

## Rule 10: Establish a long-term mentoring relationship

Finally, we suggest that you approach this mentoring experience as an open-ended one. High school students, regardless of the paths they pursue, will be embarking on profound transitions after graduation. Whether they know it or not, they could use a mentor throughout the process—for application review, general advice, and/or networking opportunities. If you have rapport, there is no reason that your mentoring relationship must end when the student stops working in the lab. However, it is possible that the student may be too shy or feel bad about asking for more of your time outside of the lab. Offer to keep in touch, and mean it. Make sure the student has a way to contact you. Follow up with their guardian, teacher, or placement coordinator 1 month or so after they have left the lab to see how they are doing. You never know the impact you can make. Good luck!

## Conclusion

Integrating a high school student into the lab has both challenges and benefits. It certainly takes time to explain concepts, teach techniques, and supervise their experiments and analyses. However, this investment has the potential to provide an invaluable opportunity for the student to engage in meaningful work and to open doors for their future educational and career opportunities.

If you are currently or soon will be a mentor to a high school student, reach out to Future PI Slack (twitter handle: @FuturePI_Slack). We have a #mentoring channel where we discuss best practices, provide advice on challenges, and share successes. We also encourage all scientists, regardless of career stage, to develop mentoring networks that they can rely on for advice, encouragement, and feedback.
